# Preoperative Balloon Aortic Valvuloplasty in a Nonagenarian With Oral Cancer and Severe Aortic Stenosis: A Case Report

**DOI:** 10.7759/cureus.103327

**Published:** 2026-02-09

**Authors:** Yasumasa Kakei, Takayoshi Toba, Takumi Hasegawa, Hiromasa Otake, Masaya Akashi

**Affiliations:** 1 Department of Oral and Maxillofacial Surgery, Kobe University Hospital, Kobe, JPN; 2 Division of Cardiovascular Medicine, Kobe University Graduate School of Medicine, Kobe, JPN; 3 Divisiont of Cardiovascular Medicine, Kobe University Graduate School of Medicine, Kobe, JPN

**Keywords:** advanced age, aortic valve stenosis, balloon aortic valvuloplasty, gingival neoplasms, mouth neoplasms, squamous cell carcinoma of head and neck

## Abstract

The concurrent presence of valvular heart disease and malignancy poses significant therapeutic challenges, particularly in patients of advanced age. Balloon aortic valvuloplasty (BAV) represents one potential option for addressing critical aortic valve disease before oncological intervention, though optimal patient selection criteria remain debated. We present a 90-year-old woman in whom echocardiographic evaluation prior to planned gingival cancer surgery revealed hemodynamically significant aortic valve narrowing. Following multidisciplinary consultation with cardiologists and anesthesiologists, BAV was performed 48 hours prior to the oncological procedure. The valve intervention produced sufficient hemodynamic improvement to permit definitive tumor resection with adequate surgical margins, including prophylactic lymph node clearance. Hospital discharge occurred approximately four weeks postoperatively without major adverse events. To the best of our knowledge, this is the first documented instance of a gingival malignancy resection following BAV. Our experience suggests that BAV may enable curative cancer surgery in carefully selected elderly patients with critical valvular disease who would otherwise face prohibitive operative risk. Nonetheless, additional evidence is necessary to define the appropriate role and safety profile of this staged therapeutic approach.

## Introduction

Oral cancer accounts for approximately 1% of all malignancies, with a male-to-female ratio of approximately 1.5:1 in Japan, indicating a higher prevalence in men [1]. The peak incidence of oral cancer in Japan has shifted to patients in their 70s, with the peak age having shifted from 70-74 years to 75-79 years; more than 55% of cases are diagnosed in patients aged 65 years or older, reflecting the ongoing trend toward an aging society [2]. Among patients with head and neck cancer, cardiovascular risk factors are highly prevalent, with hypertension being the most common comorbidity, reported in 67% of cases [3]. Among patients aged 75 or older with head and neck cancer, 75% have at least one comorbidity, with cardiovascular diseases representing a significant portion of these cases [4]. In our daily practice, older patients with oral cancer are increasingly referred to cardiologists for presurgical risk assessment for concomitant cardiovascular disease.

Aortic stenosis (AS) is a progressive narrowing of the aortic valve opening, leading to left ventricular outflow obstruction, subsequent left ventricular systolic dysfunction, and ultimately chronic heart failure [5]. It is estimated that approximately 12.4% of people aged 75 years and over have AS, corresponding to approximately 4.9 million elderly patients in Europe and 2.7 million in North America [6]. Surgical or transcatheter valve replacement (SAVR or TAVR), balloon aortic valvuloplasty (BAV), and medical therapy are important treatments for AS [7]. However, when cancer is present, the choice of these options must consider the stage of the cancer and associated treatment, expected outcomes, and comorbidities. Especially in patients undergoing non-cardiac surgery, multidisciplinary team evaluation with surgeons, cardiologists, and anesthetists is necessary because severe AS is a well-established high-risk factor for perioperative morbidity and mortality due to surgical stress and hemodynamic instability [8].

This clinical scenario presents a critical dilemma: elderly patients with coexisting severe AS and oral cancer face prohibitive cardiac risk from oncological surgery, yet delaying cancer treatment to address AS through definitive valve replacement (SAVR or TAVR) allows tumor progression. BAV has emerged as a potential bridging strategy in this context. Unlike TAVR, which requires post-procedural dual antiplatelet therapy for at least one month, BAV provides rapid short-term hemodynamic improvement without the need for prolonged antithrombotic therapy, thereby minimizing bleeding risk during subsequent cancer surgery [9]. This advantage is particularly important when timely oncological intervention is essential. Here, we report a case of a 90-year-old woman with mandibular gingival cancer who successfully underwent oral cancer surgery following preoperative BAV for severe AS.

## Case presentation

**Figure 1:**
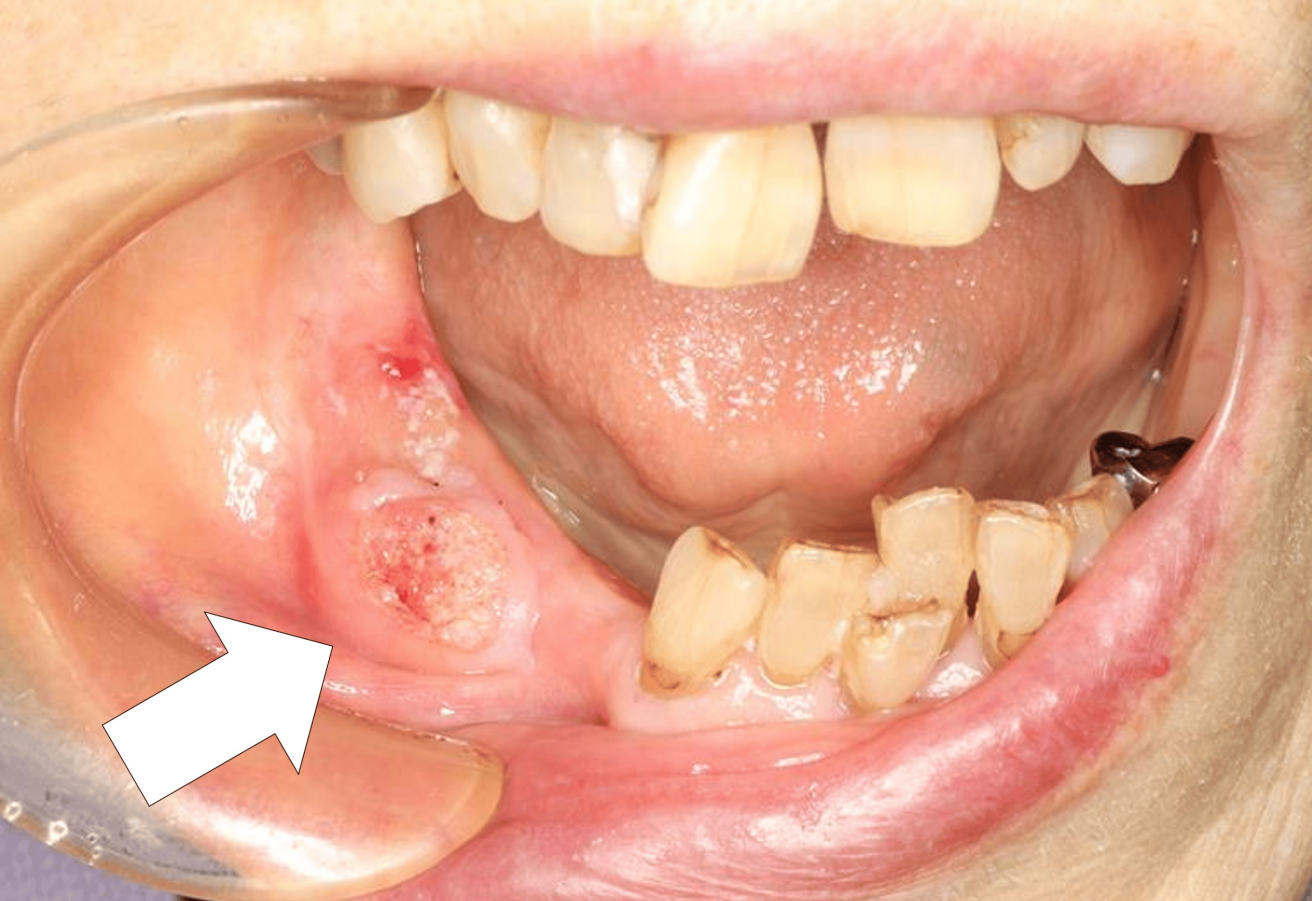
Initial clinical presentation of the mandibular gingival lesion Oral examination demonstrating an indurated mass (arrow) involving the right mandibular gingiva, measuring approximately 23 × 13 mm.

A 90-year-old woman with a clean cardiovascular history complained of pain in her lower right gingiva in September 2023. When she consulted a family dentist for denture fabrication, intraoral findings revealed a mass consolidation measuring approximately 23×13 mm, with spontaneous pain or tenderness at the right gingival margin, suspicious for mandibular gingival cancer (Figure 1).　

**Figure 2:**
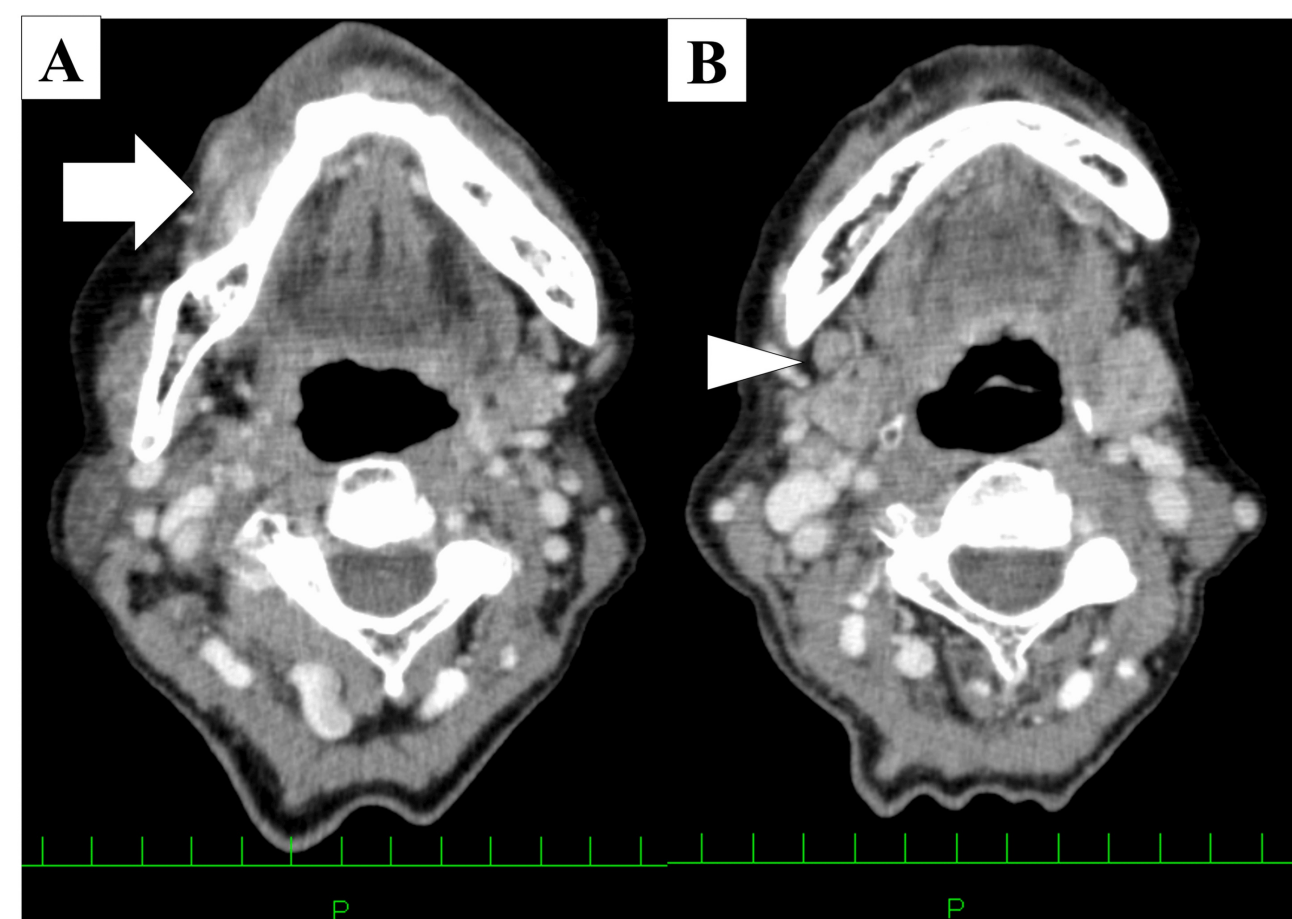
Contrast-enhanced computed tomography findings (A) Axial image showing an enhancing lesion with indistinct margins at the right gingival site (arrow). (B) Axial image demonstrating a rounded, homogeneously enhancing lymph node in the right submandibular region (arrowhead).

She was referred to the Department of Oral and Maxillofacial Surgery at Kobe University Hospital by her family dentist for further examination and treatment of a tumor in the lower right gingiva. The right submandibular lymph node was swollen, with good mobility and no palpable nodules. Contrast‑enhanced computed tomography revealed a tumor with an unclear border showing a contrast effect, and measuring 20×11 mm on the right gingiva (Figure 2A). The right submandibular cervical lymph node showed an enlarged, uniformly circular lymph node measuring 11×8 mm (Figure 2B).

**Figure 3:**
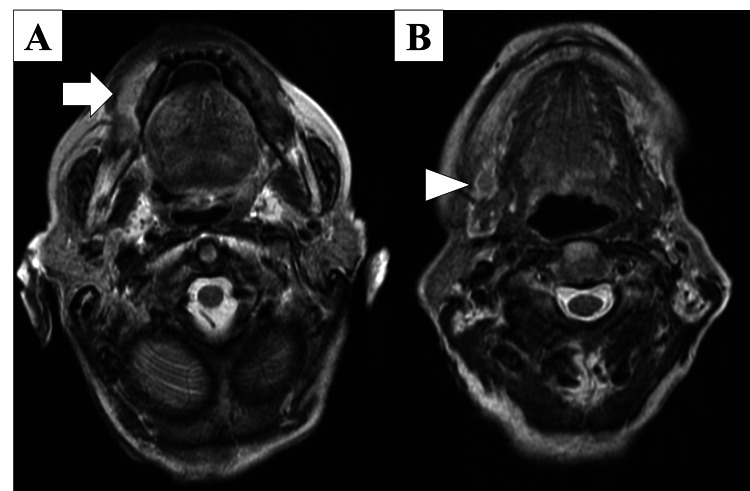
Magnetic resonance imaging findings (A) Axial T2-weighted image revealing hyperintense signal at the right gingival primary site (arrow). (B) Coronal image demonstrating signal abnormality within the right submandibular lymph node (arrowhead).

Non-contrast magnetic resonance imaging (MRI) showed a high‑signal region with a uniform internal area measuring 24×12 mm on the right side of the gingiva (Figure 3A) and a high‑signal region measuring 13×10 mm in the right submandibular cervical lymph node (Figure 3B).

**Figure 4:**
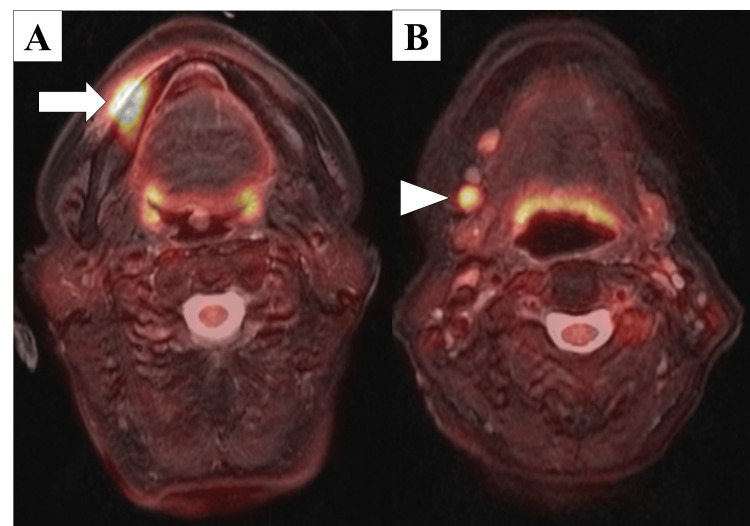
FDG-PET/MRI metabolic imaging findings (A) Axial fusion image demonstrating intense FDG avidity at the primary gingival tumor (SUVmax 18.5; arrow). (B) Coronal image showing metabolically active right submandibular lymphadenopathy (SUVmax 7.1; arrowhead). FDG-PET/MRI: 2‑[18F]-fluoro‑2‑deoxy‑D‑glucose positron emission tomography/magnetic resonance imaging

Preoperative use of 2‑[18F]-fluoro‑2‑deoxy‑D‑glucose positron emission tomography/MRI (FDG‑PET/MRI) showed abnormal FDG accumulation in the right gingiva (maximum standardized uptake value [SUVmax]: 18.5) and the right submandibular cervical lymph node (right: SUVmax: 7.1) (Figure 4).

**Figure 5:**
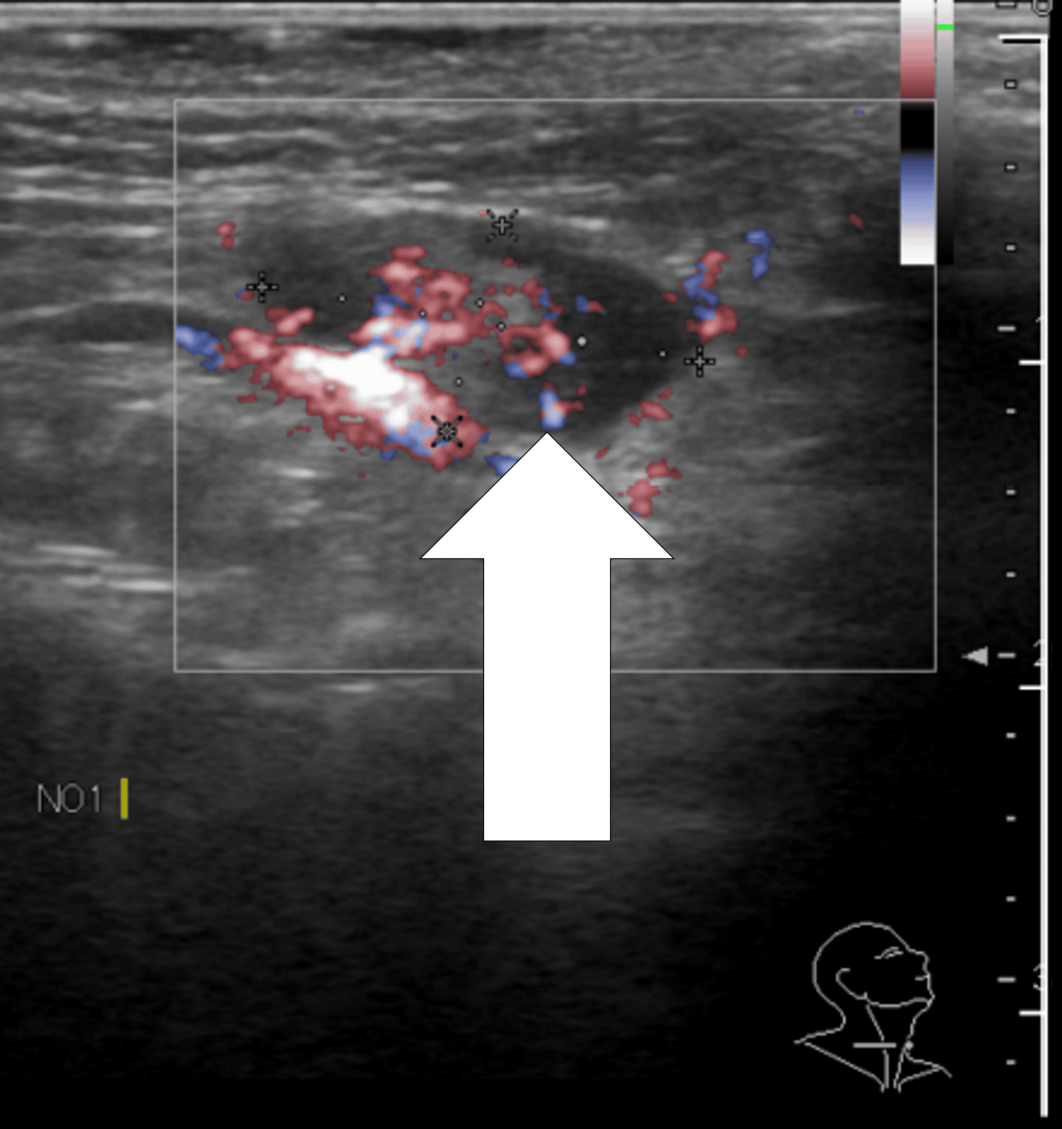
Sonographic evaluation of submandibular lymphadenopathy Ultrasound image demonstrating homogeneous internal echotexture and rounded morphology (arrow) without sonographic features of necrosis.

Ultrasonography showed that the internal structure of the lymph node was uniform, and the morphology of the lymph node was round (Figure 5).

**Figure 6:**
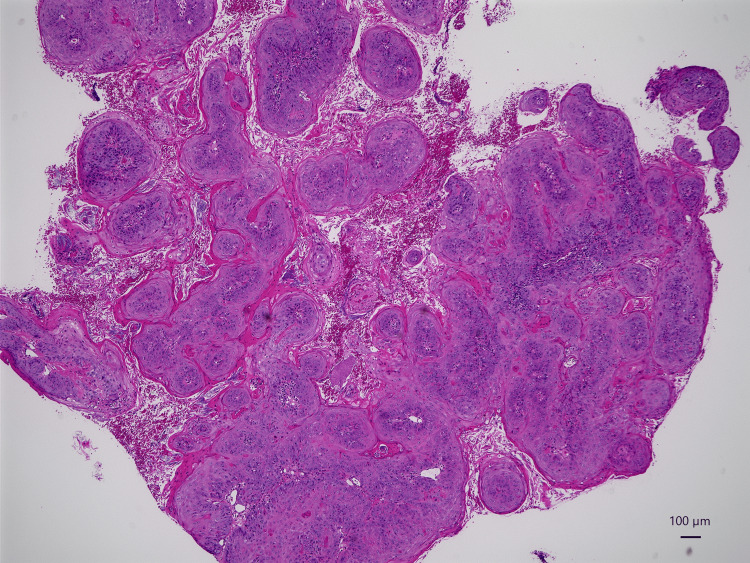
Histopathological confirmation of primary tumor Microscopic examination revealing well-differentiated squamous cell carcinoma (hematoxylin-eosin staining; magnification ×40).

Biopsy findings indicated squamous cell carcinoma (Figure 6).

The lymph nodes examined by palpation were mobile and not indurated. Following a diagnosis of stage II (cT2N0M0) mandibular gingival cancer, the patient was scheduled for a marginal mandibulectomy and neck dissection. The tumor was staged as N0 clinically by comprehensive physical assessments and various imaging findings. However, the possibility of metastasis could not be ruled out. Therefore, we performed a preventive neck dissection to address potential delayed detection of cervical metastases.

Preoperative functional assessment revealed the patient to be in New York Heart Association (NYHA) functional class II [10], with mild dyspnea on moderate exertion but no symptoms at rest. Her activities of daily living (ADL) were largely preserved; she was able to perform basic self-care activities independently but required occasional assistance with instrumental activities. Using the Clinical Frailty Scale (CFS) [11], she was assessed as CFS 4 (vulnerable), indicating she was not dependent but often complained of being "slowed up" and was mildly symptomatic. Cognitive function evaluation at an external facility revealed mild cognitive impairment. The patient had a history of hip replacement surgery. Based on these assessments, she was classified as American Society of Anesthesiologists (ASA) physical status class IV [12], and the Revised Cardiac Risk Index (RCRI) [13] was calculated as 2 points (high-risk surgery and age >70 years).

**Figure 7:**
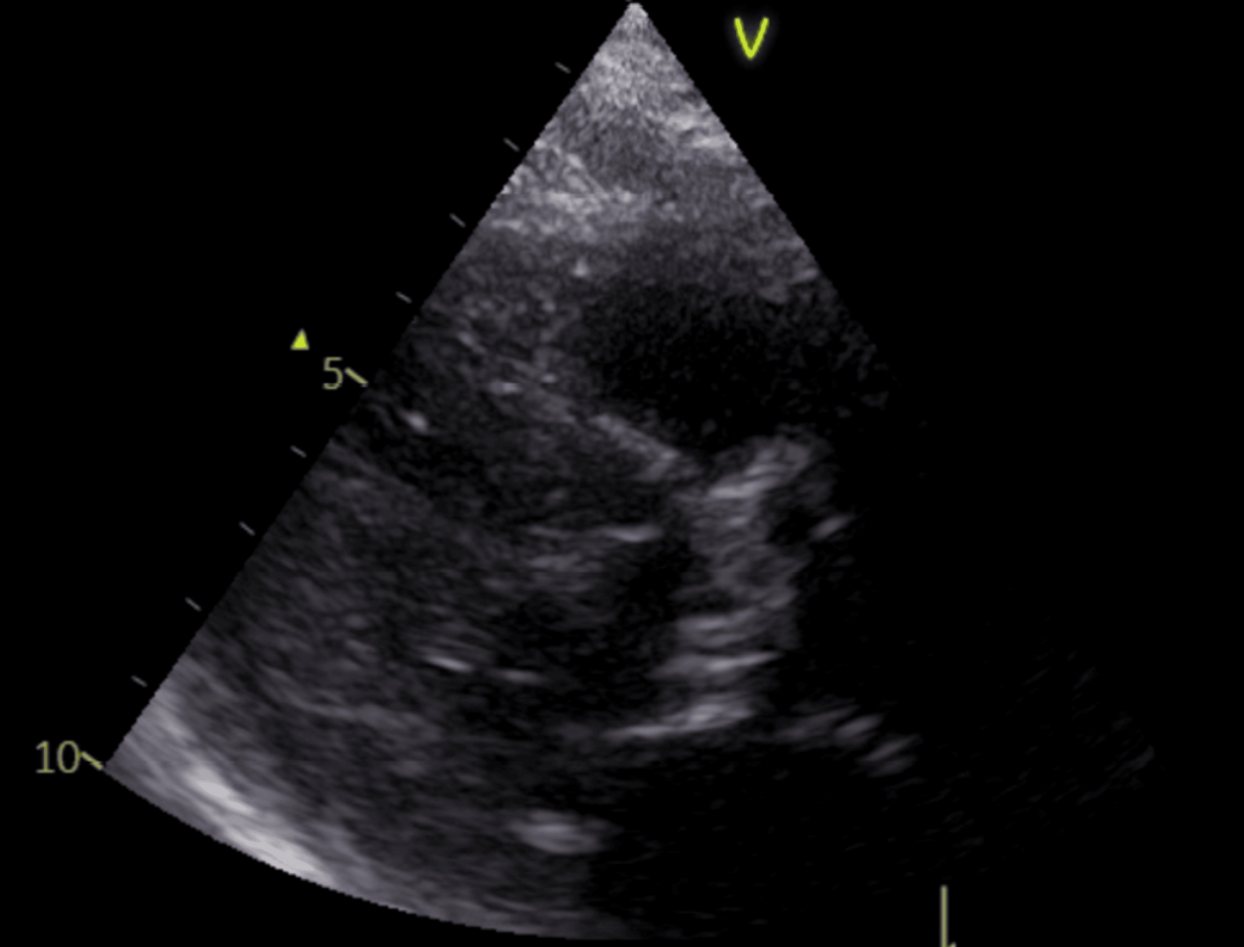
Echocardiographic assessment of aortic valve disease Parasternal long-axis view demonstrating extensive calcification of aortic valve leaflets (arrow) with peak transvalvular velocity of 4.8 m/s, consistent with severe stenosis.

A transthoracic echocardiogram showed extensive calcification of aortic valve leaflets with peak transvalvular velocity of 4.8 m/s, consistent with severe stenosis (TTE)(Figure 7).

**Figure 8:**
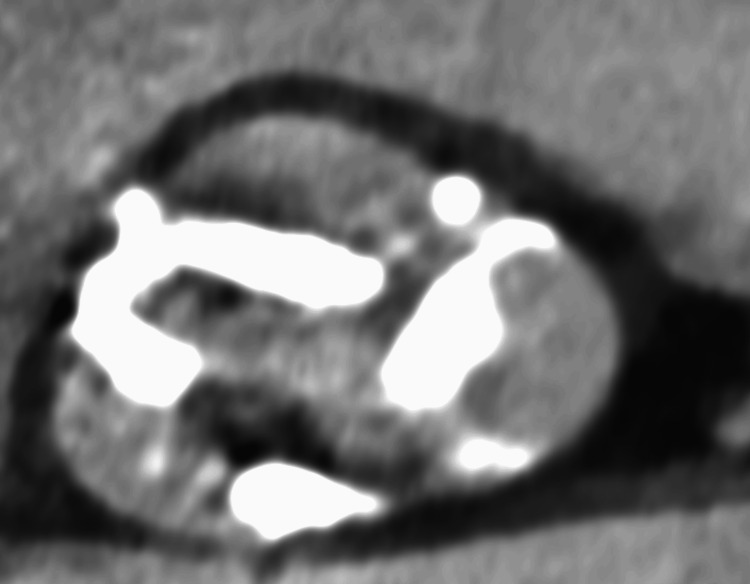
Computed tomography (CT) assessment of aortic valve calcification Severe aortic valve calcification with a symmetric tri-leaflet distribution was observed.

Preoperative computed tomography was performed to evaluate the anatomical characteristics of the aortic valve for treatment planning. The CT revealed very severe calcification with a symmetric tri-leaflet distribution (Agatston calcium score [14]: 4190 AU) (Figure 8).

The mean aortic annulus diameter was 24 mm, with a mean sinus of Valsalva diameter of 29 mm and a mean sinotubular junction diameter of 25 mm. This indicated that the valve morphology was favorable for BAV, as the symmetric calcification pattern suggested sufficient potential for controlled balloon dilatation without an excessive risk of annular rupture or severe paravalvular regurgitation. Due to near-severe aortic stenosis, elevated BNP, and long-anticipated surgery time, a multidisciplinary team advised treating the cardiac condition before proceeding with mandibular cancer surgery.

The decision to perform BAV rather than TAVR was based on multiple considerations. First, anatomically, the calcification pattern was favorable for BAV with predominantly leaflet involvement. Second, the urgent need for oncological surgery within days precluded the mandatory dual antiplatelet therapy (DAPT) period required after TAVR (typically 1-3 months). Third, the patient's extreme age and frailty status made a simpler, less invasive procedure preferable. Fourth, BAV preserved the option for future definitive TAVR after cancer treatment if clinically indicated. Considering these factors, the multidisciplinary team elected to proceed with BAV.

**Figure 9:**
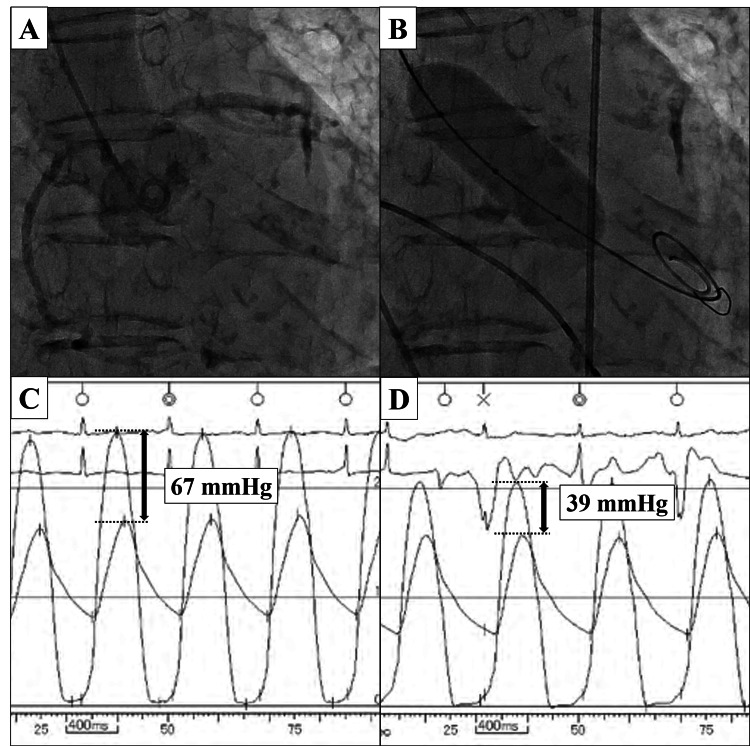
Balloon aortic valvuloplasty procedural imaging and hemodynamics (A) Pre-dilatation aortography. (B) Balloon inflation during rapid ventricular pacing. (C, D) Simultaneous left ventricular and aortic pressure recordings demonstrating reduction in transvalvular gradient from 67 mmHg to 39 mmHg (double-headed arrows).

BAV was performed with conscious sedation via the radial artery approach. The aortic valve was dilated with 18-mm TRIVAL (Kaneka Medix Corporation, Japan) under rapid pacing (200 beats/min) (Figure 9A, 9B). After four cycles of balloon dilatation, the pressure gradient between the left ventricle and aorta was improved from 67 mmHg to 39 mmHg (Figure 9C, 9D).

TTE demonstrated only a small amount of periarticular regurgitation. There were no perioperative complications related to BAV. Regarding post-BAV antithrombotic management, no antithrombotic therapy was administered after BAV.

Marginal mandibulectomy and neck dissection were performed under general anesthesia 2 days after BAV. The BAV-induced hemodynamic improvement translated into favorable intraoperative conditions. Throughout the 3-hour surgical procedure, the patient maintained hemodynamic stability without requiring vasopressor support. Mean arterial pressure remained within 65-85 mmHg, and heart rate was stable at 60-75 beats/min. No significant arrhythmias or ST-segment changes were observed on continuous electrocardiographic monitoring. Estimated blood loss was 150 ml, and no blood transfusion was required.

**Figure 10:**
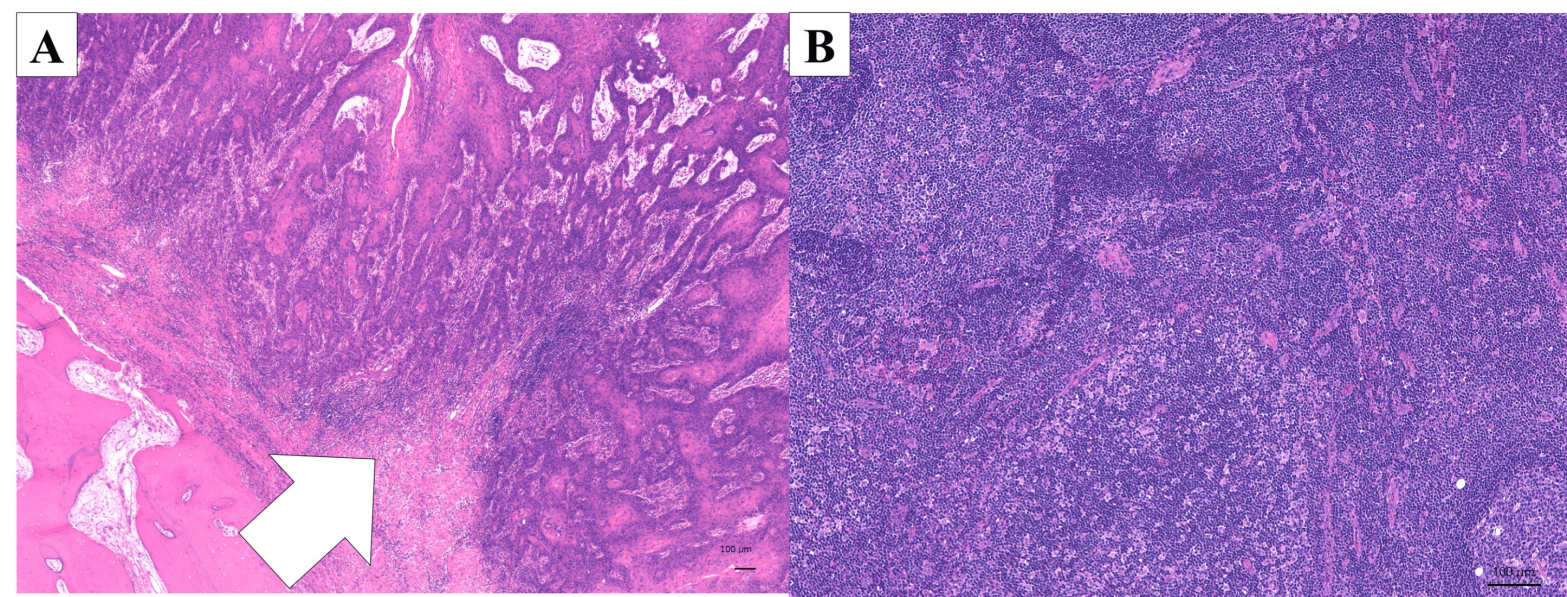
Pathological analysis of surgical specimens (A) Well-differentiated squamous cell carcinoma from primary tumor demonstrating keratinization with keratin pearl formation (hematoxylin-eosin staining; arrow). (B) Representative cervical lymph node specimen showing absence of metastatic disease (hematoxylin-eosin staining).

Histopathological findings revealed squamous cell carcinoma with prominent keratinization in a solid alveolar form (Figure 10).

Keratin pearls were occasionally found, and nuclear atypia was relatively mild. The tumor follicle measured 30×20×8 mm, and the depth of penetration was 8 mm. There were no metastases in cervical lymph nodes. The patient was discharged without complications 28 days after the mandibular gingival cancer surgery. The oral and maxillofacial surgery and cardiology teams followed up with her over two years after the surgery. There was no recurrence of mandibular gingival cancer, and no worsening of severe AS was observed. Two years after TTE, the peak flow velocity, mean pressure gradient, and aortic valve area were 4.36 m/s, 49 mmHg, and 0.29 cm², respectively (Table 1).

**Table 1:** Summary of clinical timeline, interventions, and outcomes AS, aortic stenosis; AVA, aortic valve area; BAV, balloon aortic valvuloplasty; BNP, brain natriuretic peptide; CFS, Clinical Frailty Scale; MPG, mean pressure gradient; NYHA, New York Heart Association; Vmax, peak flow velocity

Timeline	Event	Key Parameters/Outcomes
September 2023	Symptom onset	Pain in lower right gingiva
December 7, 2023	Referral to Kobe University Hospital	Tumor 23×13 mm, suspected mandibular gingival cancer
December 2023	Preoperative cardiac evaluation	Severe AS: Vmax 4.8 m/s, MPG 61 mmHg, AVA 0.41 cm²; BNP 266.44 pg/ml
Pre-BAV	Patient status assessment	NYHA Class II, CFS 4 (vulnerable), ASA Class IV
Day -2	BAV procedure	Pressure gradient reduced: 67 mmHg → 39 mmHg; No complications
Day 0	Oral cancer surgery	Marginal mandibulectomy + neck dissection; Stable hemodynamics; No vasopressors required
Day 28	Discharge	Uncomplicated postoperative course
2 years post-surgery	Follow-up	No cancer recurrence; Expected AS restenosis: Vmax 4.36 m/s, MPG 49 mmHg, AVA 0.29 cm²

## Discussion

AS is a major challenge in the management of older patients with cancer requiring surgery, as it significantly increases the perioperative risk [15]. To the best of our knowledge, this is the first documented case that demonstrates the successful use of BAV as a bridge to oral cancer surgery in a 90-year-old patient with severe AS.

Japan is currently experiencing population aging at an unprecedented rate [16]. Consequently, the number of surgical procedures performed on older patients is increasing. With this demographic shift, the incidence of both aortic valve stenosis and oral cancer is rising, and the frequency of these co-existing conditions is expected to increase [17]. The primary concern in treating oral cancer in older patients is perioperative complications, particularly cardiovascular events [18]. Therefore, at our hospital, we routinely perform preoperative echocardiography on older patients to assess cardiac function, occasionally identifying severe AS. This practice underscores the importance of evaluating the presence and severity of AS during preoperative assessment and developing appropriate treatment plans through team collaboration [19].

The management of co-existing AS and cancer requires careful consideration of several factors. SAVR remains the gold standard treatment for severe symptomatic AS according to current European Society of Cardiology guidelines, which recommend SAVR before non-cardiac surgery [7]. However, approximately 40% of patients with symptomatic AS are considered ineligible for SAVR because of advanced age, comorbidities, or active malignancy [20]. While TAVR has emerged as an alternative, the requirement for post-procedural dual antiplatelet therapy poses a significant risk of bleeding during subsequent cancer surgery [21]. For oral cancer surgery following TAVR, a waiting period of approximately 1 month is necessary [22]. During this time, oral cancer can progress rapidly, potentially necessitating more extensive reconstructive procedures such as free skin flaps [23]. However, as in our case, extended surgery may be contraindicated in very old patients, particularly those with mild dementia or previous hip replacement. Historically, such cases were often managed with best supportive care.

BAV, first introduced in 1986 [24], has evolved from its initial role as a definitive treatment to its current use as a bridge therapy. Despite the limitation of early restenosis, BAV offers several advantages in the cancer surgery setting, such as minimal invasiveness compared with SAVR or TAVR, rapid hemodynamic improvement, no requirement for long-term antiplatelet therapy, short recovery time allowing expedited cancer treatment, and the potential for future definitive AS treatment after cancer treatment [9].

The selection of BAV over TAVR in our case was based on systematic anatomical and clinical assessment. The CT-based evaluation confirmed favorable valve anatomy for BAV, with calcification predominantly involving the leaflet bodies and relative sparing of the annulus. This pattern is associated with better outcomes following balloon dilatation. Furthermore, the urgent timeline for oncological intervention, the patient's extreme age and frailty, and the desire to avoid mandatory DAPT all supported BAV as the optimal bridging strategy. It is important to acknowledge that TAVR remains a viable alternative when BAV is anatomically unsuitable, such as in cases with heavy annular calcification or unfavorable annular dimensions. In such scenarios, TAVR may be considered with modified antithrombotic regimens, though this approach requires careful balancing of thromboembolic and bleeding risks. Our case should not be interpreted as suggesting BAV is universally preferable; rather, individualized anatomical and clinical assessment is essential for treatment selection.

It is essential to acknowledge the inherently transient nature of the BAV benefit. The procedure provides immediate hemodynamic relief by mechanically fracturing calcified valve leaflets. However, restenosis typically occurs within 6-12 months as the valve leaflets re-calcify and stiffen [25]. In our case, follow-up echocardiography at two years demonstrated expected restenosis, with peak velocity increasing to 4.36 m/s, mean gradient rising to 49 mmHg, and effective orifice area decreasing to 0.29 cm². However, this anticipated hemodynamic deterioration does not negate the value of BAV as a bridging strategy. The critical window of improved hemodynamics allowed safe completion of curative oncological surgery, and the patient remained clinically stable without heart failure symptoms during the follow-up period. This underscores the importance of continued cardiac surveillance and multidisciplinary planning for potential future interventions, including TAVR, if clinically indicated.

The limitations of BAV must be recognized, including the risk of restenosis and the need for definitive valve replacement in the future [25]. For this reason, it is essential to manage AS in parallel with cancer follow-up after surgery.

Our case demonstrates that BAV can facilitate timely oral cancer surgery in older patients with severe AS who might otherwise be denied curative cancer treatment. Although this approach appears promising, careful patient selection through multidisciplinary assessment remains crucial. Further research is needed to establish optimal treatment protocols and to validate the safety and efficacy of this strategy in a larger cohort of patients.

## Conclusions

To the best of our knowledge, this is the first published case of mandibular gingival carcinoma resection following preparatory balloon aortic valvuloplasty. Our experience indicates that BAV can create a window of hemodynamic stability permitting definitive oral cancer surgery without major perioperative complications in elderly patients with critical valvular obstruction and elevated surgical risk. Nevertheless, prospective data collection and systematic analysis will be essential to establish the safety profile and identify appropriate selection criteria for this staged treatment strategy.

## References

[1] Koyama S, Tabuchi T, Okawa S, Morishima T, Ishimoto S, Ishibashi M, Miyashiro I (2020). Oral cavity cancer incidence rates in Osaka, Japan between 2000 and 2014. Oral Oncol.

[2] Fukumoto C, Ogisawa S, Tani M (2020). Clinical characteristics, treatment methods and prognoses of patients with oral squamous cell carcinoma in Japanese population: a single institution retrospective cohort study. BMC Geriatr.

[3] Sun L, Brody R, Candelieri D (2023). Risk of cardiovascular events among patients with head and neck cancer. JAMA Otolaryngol Head Neck Surg.

[4] Piccirillo JF, Vlahiotis A, Barrett LB, Flood KL, Spitznagel EL, Steyerberg EW (2008). The changing prevalence of comorbidity across the age spectrum. Crit Rev Oncol Hematol.

[5] Otto CM, Prendergast B (2014). Aortic-valve stenosis--from patients at risk to severe valve obstruction. N Engl J Med.

[6] Osnabrugge RL, Mylotte D, Head SJ (2013). Aortic stenosis in the elderly: disease prevalence and number of candidates for transcatheter aortic valve replacement: a meta-analysis and modeling study. J Am Coll Cardiol.

[7] Baumgartner H, Falk V, Bax JJ (2017). 2017 ESC/EACTS Guidelines for the management of valvular heart disease. Eur Heart J.

[8] Tashiro T, Pislaru SV, Blustin JM, Nkomo VT, Abel MD, Scott CG, Pellikka PA (2014). Perioperative risk of major non-cardiac surgery in patients with severe aortic stenosis: a reappraisal in contemporary practice. Eur Heart J.

[9] Saia F, Marrozzini C, Moretti C (2011). The role of percutaneous balloon aortic valvuloplasty as a bridge for transcatheter aortic valve implantation. EuroIntervention.

[10] New York Heart Association (1964). Diseases of the heart and blood vessels: nomenclature and criteria for diagnosis. https://pmc.ncbi.nlm.nih.gov/articles/PMC1515595/.

[11] Rockwood K, Song X, MacKnight C, Bergman H, Hogan DB, McDowell I, Mitnitski A (2005). A global clinical measure of fitness and frailty in elderly people. CMAJ.

[12] Saklad M (1941). Grading of patients for surgical procedures. Anesthesiology.

[13] Lee TH, Marcantonio ER, Mangione CM (1999). Derivation and prospective validation of a simple index for prediction of cardiac risk of major noncardiac surgery. Circulation.

[14] Agatston AS, Janowitz WR, Hildner FJ, Zusmer NR, Viamonte Jr M, Detrano R (1990). Quantification of coronary artery calcium using ultrafast computed tomography. J Am Coll Cardiol.

[15] Agarwal S, Rajamanickam A, Bajaj NS (2013). Impact of aortic stenosis on postoperative outcomes after noncardiac surgeries. Circ Cardiovasc Qual Outcomes.

[16] Muramatsu N, Akiyama H (2011). Japan: super-aging society preparing for the future. Gerontologist.

[17] Eveborn GW, Schirmer H, Heggelund G, Lunde P, Rasmussen K (2013). The evolving epidemiology of valvular aortic stenosis: the Tromsø study. Heart.

[18] Paleri V, Wight RG, Silver CE (2010). Comorbidity in head and neck cancer: a critical appraisal and recommendations for practice. Oral Oncol.

[19] Kristensen SD, Knuuti J, Saraste A (2014). 2014 ESC/ESA Guidelines on non-cardiac surgery: cardiovascular assessment and management: The Joint Task Force on non-cardiac surgery: cardiovascular assessment and management of the European Society of Cardiology (ESC) and the European Society of Anaesthesiology (ESA). Eur Heart J.

[20] Leon MB, Smith CR, Mack MJ (2016). Transcatheter or surgical aortic-valve replacement in intermediate-risk patients. N Engl J Med.

[21] Aikawa T, Kuno T, Malik AH, Briasoulis A, Kolte D, Kampaktsis PN, Latib A (2023). Transcatheter aortic valve replacement in patients with or without active cancer. J Am Heart Assoc.

[22] Wood DA, Lauck SB, Cairns JA (2019). The Vancouver 3M (Multidisciplinary, Multimodality, But Minimalist) clinical pathway facilitates safe next-day discharge home at low-, medium-, and high-volume transfemoral transcatheter aortic valve replacement centers: the 3M TAVR study. JACC Cardiovasc Interv.

[23] Shah JP, Gil Z (2009). Current concepts in management of oral cancer- surgery. Oral Oncol.

[24] Cribier A, Savin T, Saoudi N, Rocha P, Berland J, Letac B (1986). Percutaneous transluminal valvuloplasty of acquired aortic stenosis in elderly patients: an alternative to valve replacement?. Lancet.

[25] Ben-Dor I, Goldstein SA, Pichard AD (2011). Clinical profile, prognostic implication, and response to treatment of pulmonary hypertension in patients with severe aortic stenosis. Am J Cardiol.

